# Distinct roles of estrone and estradiol in endothelial colony‐forming cells

**DOI:** 10.14814/phy2.15818

**Published:** 2023-10-04

**Authors:** Alicia Ivory, Andrew S. Greene

**Affiliations:** ^1^ Department of Physiology Medical College of Wisconsin Milwaukee Wisconsin USA; ^2^ The Jackson Laboratory Bar Harbor Maine USA

## Abstract

Our current understanding of the relationship between estrogen and human endothelial colony‐forming cell (hECFC) function is based almost exclusively on studies investigating estradiol action at nuclear estrogen receptors. In the current study the hypothesis was tested that the less potent estrogen receptor agonist, estrone, affects hECFC proliferation, migration, secretion, and tube formation in a way that is unique from that of estradiol. The relationship between the estrogens, estradiol and estrone, is clinically important, particularly in postmenopausal women where estradiol levels wane and estrone becomes the predominant estrogen. Cultured hECFCs from peripheral blood mononuclear cell fractions were treated with concentrations of estradiol and estrone ranging from 1 nM to 1 μM separately and in combination. Following treatment, proliferation, migration, ability to attract other hECFCs (autocrine secretion), and ability to enhance endothelial cell tube formation (tubulogenesis) were tested. Functional assays revealed unique, concentration‐dependent physiological effects of estrone and estradiol. Estradiol exposure resulted in increased hECFC proliferation, migration, secretion of chemoattractant, and enhancement of tube formation as expected. As with estradiol, hECFC secretion of chemoattractant increased significantly with each increase in estrone exposure. Estrone treatment produced a biphasic, concentration‐dependent relationship with proliferation and tube formation and relatively no effect on hECFC migration at any concentration. The quantitative relationship between the effects of estrone and estradiol and each hECFC function was analyzed. The extent to which estrone was similar in effect to that of estradiol was dependent on both the concentrations of estradiol and estrone and the hECFC function measured. Interestingly, when the two estrogens were present, differing ratios resulted in unique functional responses. hECFCs that were treated with combinations of estrone and estradiol with high estrone to estradiol ratios showed decreased proliferative capacity. Conversely, hECFCs that were treated with combinations that were relatively high in estradiol, showed increased proliferative capacity. Cells that were treated with estrone and estradiol in equal concentrations showed an attenuated proliferative response that was decreased compared to the proliferation that either estrone or estradiol produced when they were present alone. This co‐inhibitory relationship, which has not been previously reported, challenges the prevailing understanding of estrone as solely a weak agonist at estrogen receptors. This study provides evidence that estrone signaling is distinct from that of estradiol and that further investigation of estrone's mechanism of action and the biological effect may provide important insight into understanding the dysfunction and decreased number of hECFCs, and the resulting cardiovascular disease risk observed clinically in menopausal women and women undergoing hormone replacement therapy.

## 1 INTRODUCTION

Human endothelial colony‐forming cells (hECFCs) are a stem cell population that circulates in the peripheral blood and supports endothelial functions of proliferation, migration, and angiogenesis (Banno & Yoder, 2018; Ferratge et al., 2017; Medina et al., 2017; Tasev, Koolwijk, & van Hinsbergh, 2016). Only recently has this cell population been recognized as a unique subset of endothelial progenitor cells (EPCs) that is responsible for many of the beneficial effects ascribed to EPCs (Medina et al., 2017). HECFCs are defined here as peripheral blood‐derived stem cells that express surface markers CD34, VEGFR‐2, CD133, FLK1, and aldehyde dehydrogenase antigen (Keighron et al., 2018; Medina et al., 2017; Tasev, Konijnenberg, et al., 2016; Yu et al., 2018). Increased hECFC number correlates with increased flow mediated dilation and decreased cardiovascular disease risk (Bitterli et al., 2016; Jialal et al., 2010; Werner et al., 2007). Estrogens have been shown to have a vascular protective role in premenopausal women through their action on endothelial cells, myocardium, smooth muscle cells, and hECFCs (Knowlton & Lee, 2012; Matsubara & Matsubara, 2012). The benefit of estrogen with respect to vascular cell types, including hECFCs, is lost over the menopausal transition with decreased ovarian function and estrogen production (Topel et al., 2017). This results in impaired flow mediated dilation and increased cardiovascular risk post‐menopause (Stanhewicz et al., 2018).

Studies investigating the mechanism of estrogen's protective role on enodthelial colony forming cells (ECFCs) have focused on the effects of the estrogen that is most prominent in premenopausal women, estradiol (Bulut et al., 2007; Lieberman et al., 1994; Moreau, 2019). Mechanistic studies of estradiol and hECFC function have primarily concentrated on estradiol action on hECFCs through nuclear estrogen receptors (Trenti et al., 2018). In vitro studies have shown that estradiol increases ECFC proliferation, migration, and tube formation (Blum, 2015; Rudzitis‐Auth et al., 2016; Yuan et al., 2018; Zhao et al., 2008). The actions of other prominent estrogens have not been investigated.

In vivo studies of estrogen replacement revealed the importance of understanding the functional impact of estrogen composition (the types of estrogens present in the plasma) (Cagnacci & Venier, 2019; Comhaire, 2016; Friel et al., 2005; Lobo, 2017; Machens, 2003). In premenopausal women, plasma estradiol levels range from 30 to 400 pg/mL (approximately 0.1–1 nM) and increase in estradiol concentration throughout the lifespan and with menstrual cycle variation have been shown to correlate with increased hECFC number and increased vascular function. This positive correlation exists in both humans and animal models throughout the menstrual cycle and through the menopausal transition or following ovariectomy (da Silva et al., 2016; Strehlow et al., 2003). After ovarian estrogen production decreases, hECFC number and vascular function decline. Estrogen replacement therapy restores physiological estradiol levels but has been shown not to restore hECFC number or consistently reduce cardiovascular risk. Thus, understanding the decrease in vascular function concurrent with the loss of ovarian function requires investigation of the actions of other estrogens.

A noteworthy change that occurs over the menopausal transition and throughout estrogen replacement therapy is the composition of unconjugated estrogens in the plasma. Prior to menopause, estradiol is the dominant estrogen and exists at a ratio of approximately 3:2 with its precursor, estrone (plasma levels of approximately 0.03–0.75 nM), a weaker agonist at nuclear estrogen receptors (Bhavnani & Stanczyk, 2014; Coburn et al., 2019). With hormone replacement therapy using conjugated equine estrogens or oral estradiol, estrone concentrations increase, further expanding the perturbed estrone/estradiol ratio (Rezvanpour & Don‐Wauchope, 2017; Santoro et al., 1996).

Several clinical studies have investigated health outcomes with respect to estrone in the context of cardiovascular disease revealing that the cardiovascular effects of estrone are complex and concentration dependent. High estrone levels have been associated with carotid echogenicity, an indicator of carotid calcification (Silva et al., 2008). Low levels of estrone have been linked to all‐cause mortality in postmenopausal women with coronary artery disease (de Mansur et al., 2012). In one study, high levels of estrone were found to negatively impact myocardial flow after infarction (Dong et al., 2014). Interestingly, high levels of estrone were also found to be associated with increased flow mediated dilation of the brachial artery in postmenopausal women with cardiovascular disease (Thurston et al., 2018). Few basic studies have explored estrone's actions outside of its function as an antagonist of estradiol through competitive binding at nuclear estrogen receptors. However, further investigation of how and to what extent estrone affects the cardiovascular system is necessary to better understand the mechanisms that underlie postmenopausal changes in cardiovascular risk as well as the risks and benefits of estrogen‐based treatments.

In this paper, the hypothesis that estrone and estradiol each have unique and distinct effects on the function of hECFC was tested. Using a set of functional assays measuring hECFC proliferation, migration, tube formation, and chemoattractant secretion, distinct function‐specific hECFC responses to estrone, estradiol and the two estrogens in combination were elucidated.

## 2 METHODS

### 2.1 Cell culture

hECFCs were purchased from Celprogen (Celprogen 36053‐5, CD34, VEGFR‐2, or AC 133 (CD133), CD117/ ckit, VEGFR2/ KDR/ FLK‐1, and aldehyde dehydrogenase antigen‐positive) and were routinely cultured in Celprogen's Human Endothelial Progenitor Complete Growth Medium (M36053‐05S) in T‐25 Celprogen extracellular matrix coated flasks (E36053‐05‐T25) and incubated at 37°C in the presence of 5% CO_2_. In all studies, hECFCs were sub‐cultured (Passages 3–6) into Celprogen serum free medium (M36053‐05E) for 12 h before being treated or used in functional assays.

### 2.2 Proliferation assay

5 × 10^4^ serum starved hECFCs were seeded into 24 well plates and incubated with IncuCyte® NucLight Rapid Red Reagent for nuclear labeling (Cat. No. 4717). Celprogen media with serum and antibiotics was supplemented with the desired treatment. Treatment groups included: estrone, estradiol, or 1 to 1 ratio combination of estrone/estradiol at 0 nM (medium control), 1 nM, 10 nM, 100 nM, and 1 μM. The hECFCs were then incubated for 24 h during which the cells were imaged in the IncuCyte SX5 Live‐Cell Analysis System (Sartorius). Cell number was then quantified and presented as percent of control.

### 2.3 Transwell migration assays

Cell migration was determined using Transwell culture inserts (Corning CLS3401‐48EA according to the manufacturer's instructions. In brief, 1 × 10^3^ hECFCs that were treated for 24 h with estrone and/or estradiol (grouped as described above) and seeded onto the upper chamber of each well and allowed to migrate toward the lower chamber containing 500 μL of conditioned medium taken from control ECFCs. Transwells were incubated at 37°C, 5% CO_2_, for 2 h. Migrated cells were then fixed using 4% paraformaldehyde, stained with DAPI, imaged in the center of the well using florescent microscopy at 10×, and counted using pipeline v.5 software (Prisco et al., 2014). Migration was expressed as the percent of cells that migrated in the experimental well compared to control wells treated with medium alone.

### 2.4 Paracrine chemoattractant assay

Paracrine function was determined using a cell migration assay with conditioned media as chemoattractant. 1 × 10^4^ hECFCs were treated for 24 h (groups described above), medium was then removed, fresh serum free Celprogen medium was added, and the cultures were incubated for eight additional hours. After incubation, the medium taken from the treated cells was considered “conditioned media” and used as chemoattractant in a migration assay using untreated cells as the migrating cells. 1 × 10^3^ cells/well of untreated hECFCs were seeded onto the upper chamber and allowed to migrate toward the lower chamber containing only the conditioned medium. Transwells were incubated at 37°C, 5% CO_2_, for 4 h. Migrated cells were fixed, DAPI stained, imaged and counted (as described above). Migration was then expressed as percent of cells migrated compared to control (medium from untreated cells).

### 2.5 Tube formation assay

hECFCs were incubated in human endothelial progenitor cell medium (Celprogen catalog #M36053‐05DS) on fibronectin coated 6‐well plates until the cells adhered and unrolled. Cells were then incubated in human endothelial progenitor cell serum free media for 24 h. After the serum starved period, hECFCs were incubated in human endothelial progenitor cell medium supplemented with estrone or estradiol (groups described above) for 24 h. Cells were then lifted using 0.05% trypsin and co‐cultured with human microvascular endothelial cells in 15 well Ibidi Angiogenesis Plates (Ibidi catalog #81506) on Geltrex extracellular matrix (Thermo Fisher catalog #A1413302). 2 × 10^3^ microvascular endothelial cells were cultured with 5 × 10^2^ hECFCs. Brightfield images were taken at 10× magnification of the entire well at 12 h incubation and tube length was quantified using Pipeline A1413302 v1.5 software (Prisco et al., 2014).

### 2.6 Statistical analysis

All results are expressed as mean +/− SD. Statistical significance was evaluated using an unpaired Student *t*‐test for the comparison between two groups and ANOVA followed by Fisher's post hoc test for the comparison among multiple groups. Comparisons were run for the control and treatment groups and multiple comparisons were run between each treatment group. A probability value less than 0.05 was interpreted to denote statistical significance.

## 3 RESULTS

**FIGURE 1:**
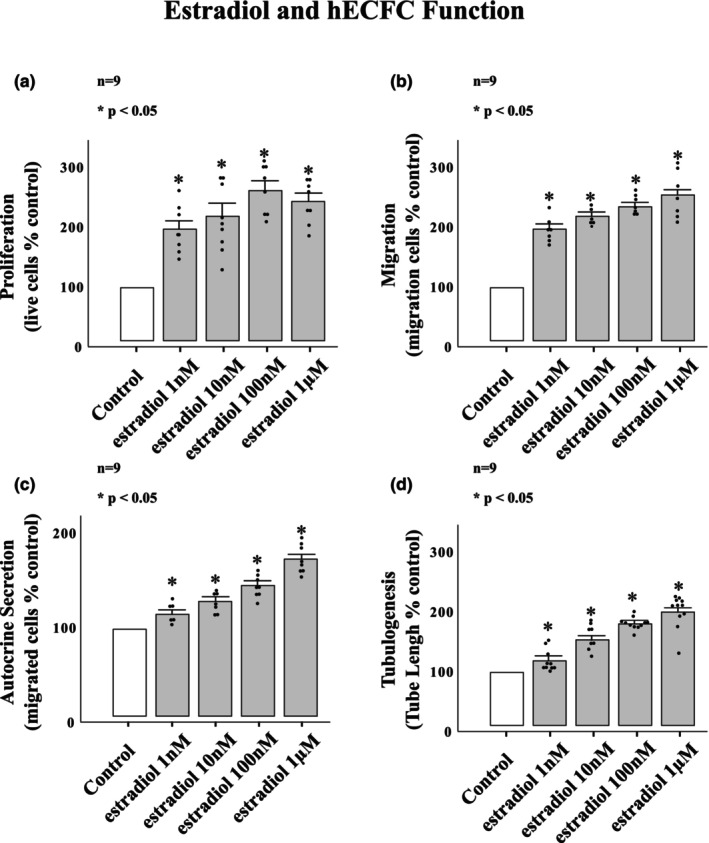
hEPC functional response to estradiol. Estradiol enhanced hEPC (a) proliferation, (b) migration, (c) autocrine secretion, and (d) tubulogenesis significantly above that of control. Estradiol ability to enhance hEPC function was consistent across all tested functions. No concentrations of estradiol were found to be functionally inhibitory to hEPCs. *p < 0.001.

Figure 1 represents the effects of a range of estradiol exposures on hECFC proliferation, migration, autocrine secretion, and tubulogenesis. All four of the tested hECFC functions were augmented by exposure to estradiol. Augmented hECFC proliferation was demonstrated beginning at estradiol concentrations of 1 nM with further significant increases in proliferation with exposure to estradiol at 10 and 100 nM. Proliferative response to estradiol at 1 μM did not significantly enhance hECFC proliferation over that of estradiol at 100 nM on post hoc analysis, resulting in a plateau in response (Figure 1a). Migration was significantly increased compared to control by exposure to estradiol at concentrations of 1 nM, 10 nM, 100 nM, and 1 μM (Figure 1b). Autocrine secretion (the ability to secrete chemotactic factors that attract other hECFCs) was significantly increased by estradiol exposures of 1 nM, 10 nM, 100 nM, and 1 μM. On post hoc analysis, each tenfold increase in estradiol concentration resulted in significant increases in autocrine secretory function (Figure 1c). Tubulogenesis was also increased consistently and significantly with 10‐fold estradiol exposure increases and plateaued at 100 nM with nonsignificant increase between 100 nM and 1 μM on post hoc analysis. Estradiol exposures of 1 μM did not result in a significant increase in tubulogenesis over that OD 100 nM (Figure 1d).

**FIGURE 2:**
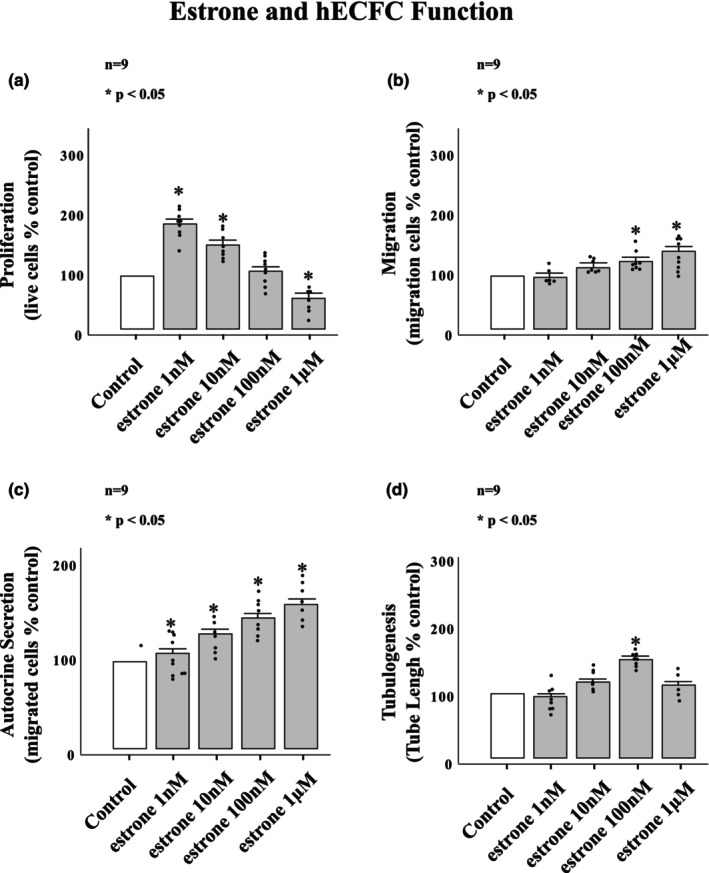
hEPC functional response to estrone. Estrone exposure had a parabolic relationship with hEPC (a) proliferation and (d) tubulogenesis. (b) Estrone was no effect on migration at 1 and 10 nM but had a small positive effect on hEPC migration at 100 nM and 1 μM. (c) Estrone exposure significantly enhanced autocrine secretion at 10 nM, 100 nM and 1 μM above that of control. *p < 0.001.

Figure 2 represents the functional effects of estrone on hECFCs at estrone concentrations of 1 nM, 10 nM, 100 nM, and 1 μM. The way in which estrone exposure affected hECFC proliferation, migration, autocrine secretion, and tube formation was unexpected and different for each function. The effect of estrone on hECFC proliferation was biphasic with only exposures of 1 nM and 10 nM resulting in significant increases in hECFC proliferation (Figure 2a). The proliferative response was maximal with 1 nM estrone exposure on post hoc analysis. An exposure of 10 nM resulted in significant increases above that of control but with significantly less than the proliferative results of estrone exposure of 1 nM. Estrone exposure at 1 μM resulted in attenuated proliferation and resulted in a significantly lower hECFC cell number than control. Estrone's effect on migratory function of hECFCs was distinct from that of the effect on proliferation. Only the highest estrone concentrations resulted in increased migration and that effectg was very small (Figure 2b). As seen with estradiol, estrone exposure increased autocrine secretion (hECFC ability to attract control hECFCs) in a concentration‐dependent manner with no observed inhibitory concentration. Interestingly, at low concentrations, estrone enhanced hECFC autocrine secretion in a way that was both qualitatively and quantitatively similar to that of estradiol. A plateau was reached at 100 nM and autocrine secretion was not further enhanced with estrone exposure of 1 μM on post hoc analysis. Estrone exposure affected hECFC enhancement of endothelial cell tube formation in a parabolic manner, where significant enhancements of tube formation were only observed at 100 nM and not observed in 1 nM, 10 nM or 1 μM concentrations (Figure 2d).

**FIGURE 3:**
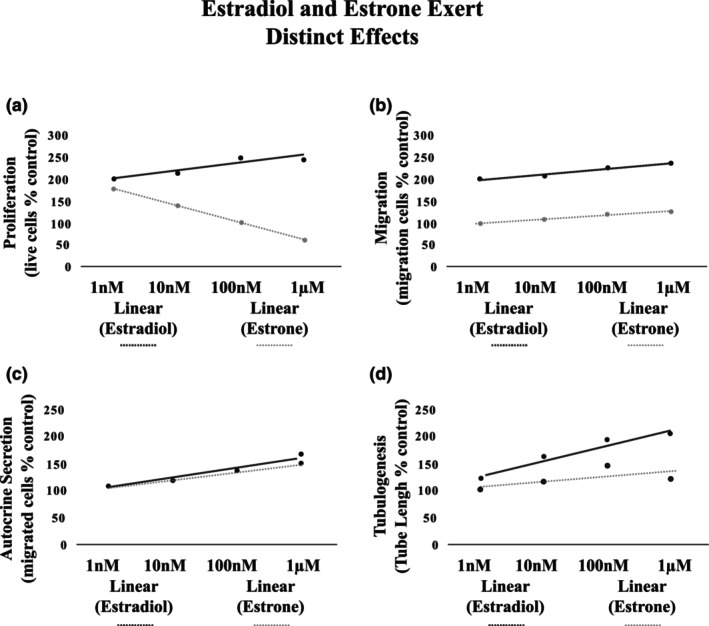
The relationship between hEPC function and estrone concentration is distinct from the relationship between hEPC function and estradiol concentration. Analysis of the trendlines of dose‐response to estrogen revealed that the comparison between the functional effects of estradiol and those of estrone are both dose and function dependent. (a) The dose response relationship of estrone and estradiol proliferation were very different. With increasing concentration, the effects of estradiol and estrone become increasingly divergent. The trends in the effects of estradiol and estrone for the functions of migration (b), autocrine secretion (c) and tubulogenesis (d) were similar in that increasing doses increased the observed functional response for both estrogens. The differences in the response observed in proliferation compared do that of the other tested functions is likely due to activation of distinct estrogen pathways by each estrogen.

The data from each functional assay were grouped by concentration and plotted on a dose–response curve that represented the response of hECFCs to increasing concentrations of estrone and estradiol. The linear approximation of the dose–response curves for estrone and estradiol were plotted together in Figure 3 to aid in visualizing and comparing the functional effects of estrone to the functional effects of estradiol. This figure shows that the relationship between the effects of the two estrogens is concentration‐dependent and function specific. The functional relationship between estrone and hECFC migration (Panel b), autocrine secretion (Panel c), and tubulogenesis (Panel d) were qualitatively similar, meaning that they each enhance hECFC migratory, secretory and tubulogenic function, to some extent, with increasing concentration (the trend line for each function is positively sloped). The effects of estrone and estradiol on hECFC proliferation were not qualitatively similar. Although, they were positively correlated at 1 nM estrogen concentrations, at higher concentrations, the effects diverged with exposure to estrogen at 10 nM, 100 nM, and 1 μM.

**FIGURE 4:**
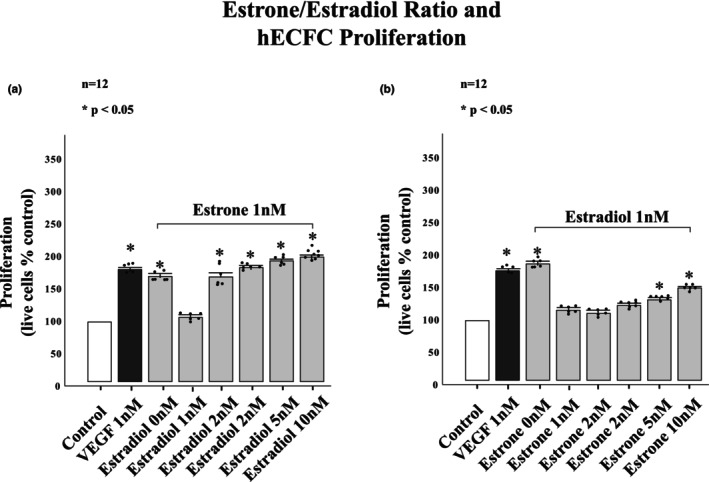
Differential effects of estrone/estradiol ratios on hECFC proliferation. (a) High estrone to estradiol ratios did not significantly enhance hECFC proliferation in ratios of 2 to 1 or 3 to 1. Ratios of 5 to 1 and 10 to 1 significantly enhanced hECFC proliferation but did not enhance proliferation to the extent of estradiol administered in 1 nM concentration alone. (b) Low estrone to estradiol ratios (ratios of 1 nM of estrone was administered with 1, 2, 3, 5, and 10 nM of estradiol enhanced hECFC proliferation significantly. This data shows that estrone and estradiol are co‐inhibitory and distinct in their ability to inhibit each other. *p < 0.001.

The contrasting effects of estrone and estradiol on hECFC proliferation were investigated further by treatment of estrone and estradiol in combination using ratios of 1 to 1, 2 to 1, 3 to1, 5 to 1, and 10 to1. Figure 4 shows the results of the combined effects of the estrone to estradiol ratio. hECFCs treated with 1 nM of estrone and increasing concentrations of estradiol showed proliferative responses for all combinations except 1 to1. Ratios of 1 nM estrone with 2, 3, 5, and 10 nM of estradiol (Panel a) (low estrone/estradiol ratio) showed significant increases in proliferation compared to control. hECFCs that were treated with 1 nM of estradiol showed decreased proliferative capacity when treated with 2, 3, 5, and 10 nM of estrone (Panel b) (high estrone/estradiol ratio).

**FIGURE 5:**
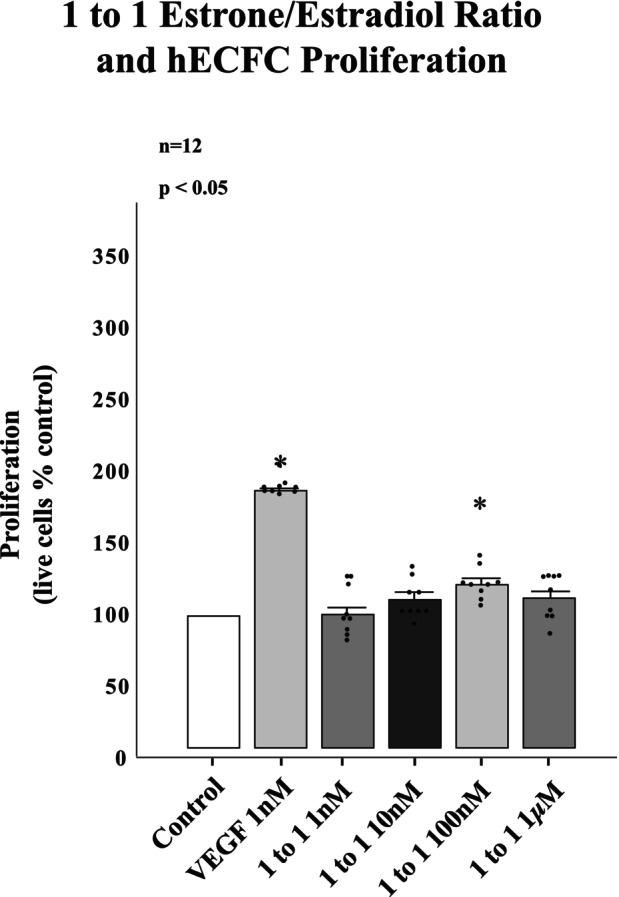
The Ratios of 1 to 1 of estrone to estradiol result in little enhancement of hECFC proliferation. Administration of the combination of estrogens at 1 nM, 10 nM, and 1 μM were unable to significantly enhance hECFC proliferation. *p < 0.001.

Further investigation of the proliferative effects of estrone to estradiol ratios in 1 to 1 combination revealed no significant proliferative response at concentrations of 1 nM, 10 nM, 100 nM or 1 μM (Figure 5).

## 4 DISCUSSION

This is the first study to examine the effects of estrone and its interaction with estradiol on hECFC function. The principal finding of this study is that estradiol and estrone exert distinct effects on hECFCs that are dependent on both the concentration of estrogen exposure and the hECFC function that was assessed. When functional effects of both estrogens were examined independently, estrone and estradiol were shown to exert enhancing effects that were qualitatively similar for the functions of migration, autocrine secretion, and tubulogenesis. However, these data showed that although both estrogens enhanced effects on hECFC proliferation at low concentrations, their effects diverge at concentration of 10 nM, 100 nM, and 1 μM (Figure 3). This divergence in effect is inconsistent with the hypothesis that these two estrogens are acting solely as agonists and partial agonists of the same receptors that then result in activation of the same pathways and yield the same functional results. These data instead, support the concept that estrone and estradiol likely act with some variability on the same receptors, that then result in differential activation of intracellular pathways and yield distinct and intricately controlled biological outcomes.

Estradiol has been shown in many studies to enhance hECFC proliferation, migration, adhesion, secretion of VEGF, and angiogenic functions. Consistent with those findings, estradiol significantly enhanced all four of the hECFC functions that were tested. The enhancement of hECFC migration and autocrine secretion was observed at all tested estradiol concentrations.

Like thyroid hormone, estradiol and other estrogens regulate numerous intracellular mechanisms that modulate their biological effects (Davis et al., 2016). Proliferation of ECFCs in response to estradiol, is a complex response that likely results from the summation of many converging pathways. For example, notch (Chen et al., 2012), WNT (Batsali et al., 2017; Pavlaki et al., 2014), and TGFβ (Katoh, 2013; Sales et al., 2006) pathways have all been shown to regulate ECFC proliferation both independently and in combination. One putative mechanism by which the net proliferative response of the combination of estrogens is regulated may be through coordinated modulation of groups of pathways such as these.

This study found that estrone, at equal concentrations to estradiol, significantly attenuated the proliferative effects of estradiol on hECFCs and further, that estrone exposure was sufficient to significantly suppress the proliferative effects of estradiol when present in 1 to 1, 2 to 1, 3 to 1, 5 to 1 and 10 to 1 concentration ratios. Estrone, in ratios of 1 to 1, 2 to 1, and 3 to 1 with 1 nM of estradiol attenuated the proliferative response to 1 nM of estradiol completely. Only estrone to estradiol ratios that were 5 to 1 and 10 to 1 enhanced hECFC proliferation over that of control. This is interesting because concentrations of estrone at 1 and 10 nM significantly enhanced proliferation when estradiol was not present. Ratios of estrone to estradiol, where estradiol was present in higher concentrations, induced hECFC proliferative responses that were comparable to the effects that estradiol could induce when present alone. This is evidence of a complex co‐inhibitory relationship between estrone and estradiol. This co‐inhibition was also evident when cells were treated with estrone and estradiol in 1 to 1 combination at increasing concentrations. The 1 to 1 combination at every cumulative concentration was unable to enhance proliferation over that of control.

The physiological effects of estrone exposure have not previously been studied in hECFCs. Estrone, as a partial agonist of ERα and ERβ, is presumed to affect hECFC proliferation, migration, autocrine secretion, and tube formation in a manner that is qualitatively like that of estradiol. The data presented in this study refute that hypothesis and suggest a more complex interaction. Correlation analysis of the effects of estrone exposure and estradiol exposure revealed divergent effects. This is an essential point since both the physiological and pathophysiological implications of estrogen and estrogen signaling are far reaching. Estrogen has key roles in the normal development of males and females, as well as in the regulation of fertility, bone health, cancer, and as emphasized here, vascular function and cardiovascular disease. Throughout the female lifespan, and in the setting of hormone replacement therapy, the sizable changes in estrogen concentration (mostly estradiol and estrone) and composition (estrone/estradiol ratio) have been associated with cardiovascular disease phenotypes that have been unexplained by the clinical and the scientific communities. The current study provides some insight into the complexities of the relationship between estrogen in vascular function and highlights the possible contributions of both the estrone concentration and the estrone to estradiol ratio in hECFC function.

Although this study did not resolve the specific intracellular mechanisms involved in the interplay between estrone and estradiol signaling, it is the first such study to carefully describe this interaction at the level of several essential cellular phenotypes in hECFCs. Estradiol exerts its physiological effects through complex genomic, non‐genomic, and epigenomic pathways (Clark et al., 1979; Murphy, 2011; Saczko et al., 2017). Estrone, a known partial agonist at nuclear estrogen receptors, has been shown to result in the activation of similar pathways to that of estradiol (Rauschemberger et al., 2008). However, the literature is devoid of studies that investigate estrone action on other estrogen‐responsive pathways such as those activated through membrane‐bound estrogen receptors, truncated nuclear estrogen receptors such as ERα‐36 or G protein‐coupled estrogen receptor (GPER). These pathways are involved in estrogen action and as such, are good candidates for further investigation of mechanisms by which estrone and estradiol co‐inhibit their respective actions.

## 5 CONCLUSION

This study provides evidence that estrone has distinct and dose‐dependent effects on hECFC function compared to estradiol. Further, the estrone to estradiol ratio was shown to be as important factor as the estrogen concentration in determining hECFC proliferation, a clinical indicator of cardiovascular health. This finding has significance in the clinical context of postmenopausal hECFC decline and provides insight into the role of the contribution of estrogens in cardiovascular disease.

## AUTHOR CONTRIBUTIONS

Alicia Ivory was involved in study design, experimental design, conducted experiments, and wrote manuscript. Andrew S. Greene was involved in study design, experimental design, and wrote manuscript.

## ETHICS STATEMENT

The authors have no conflicts to disclose.

## FUNDING INFORMATION

Not applicable.
